# Predictors of treatment failure for adenocarcinoma *in situ* of the uterine cervix: Up to 14 years of recorded follow-up

**DOI:** 10.3892/ol.2022.13477

**Published:** 2022-08-25

**Authors:** Karen Belkić, Sonia Andersson, Susanna Alder, Miriam Mints, David Megyessi

**Affiliations:** 1 Department of Oncology-Pathology, Karolinska Institute, SE-17176 Stockholm, Sweden; 2 School of Community/Global Health, Claremont Graduate University, Claremont, CA 91711, USA; 3 Institute for Health Promotion and Disease Prevention Research, Keck School of Medicine, University of Southern California, Los Angeles, CA 90032, USA; 4 Department of Women's and Children's Health, Obstetrics-Gynecology Division, Karolinska Institute, Stockholm, SE-17176 Stockholm, Sweden; 5 Department of Gynecology and Obstetrics, School of Medical Sciences, Faculty of Medicine-Health, Örebrö University, SE-70182 Örebrö, Sweden

**Keywords:** adenocarcinoma-*in-situ*, treatment failure, margin status, papillomavirus infection

## Abstract

The incidence of adenocarcinoma-*in-situ* (AIS) of the uterine cervix is rising, with invasive adenocarcinoma becoming increasingly common relative to squamous cell carcinoma. The present study reviewed a cohort of 84 patients first-time treated by conization for histologically-confirmed AIS from January 2001 to January 2017, to identify risk factors associated with recurrent/persistent AIS as well as progression to invasive cervical cancer. Nearly 80% of the patients were age 40 or younger at conization. Endocervical and ectocervical margins were deemed clear in 42 of the patients. All but two patients had ≥1 follow-up, with post-conization high-risk human papilloma virus (HPV) results documented in 52 patients. Altogether, 12 histopathologically-confirmed recurrences (14.3%) were detected; two of these patients had microinvasive or invasive carcinoma. In three other patients cytology showed AIS, but without recorded histopathology. Eight patients underwent hysterectomy for incomplete resection very soon after primary conization; they were not included in bivariate or multivariate analyses. Having ≥1 post-follow-up positive HPV finding yielded the highest sensitivity for histologically-confirmed recurrence: 87.5 [95% confidence interval (CI) 47.4-99.7]. Current or historical smoking status provided highest specificity: 94.4 (95% CI 72.7-99.9) and overall accuracy: 88.0 (95% CI 68.8-97.5) for histologically-confirmed recurrence. With multiple logistic regression (MLR), adjusting for age at conization and abnormal follow-up cytology, positive HPV18 was the strongest predictor of histologically-confirmed recurrence (P<0.005). Having ≥2 positive HPV results also predicted recurrence (P<0.02). Any unclear margin yielded an odds ratio 7.21 (95% CI 1.34-38.7) for histologically-confirmed recurrence adjusting for age, but became non-significant when including abnormal cytology in the MLR model. The strong predictive value of HPV, particularly HPV18 and persistent HPV positivity vis-à-vis detected recurrence indicated that regular HPV testing for patients treated for AIS is imperative. In conclusion, furthering a participatory approach, including attention to smoking with encouragement to attend needed long-term follow-up, can better protect these patients at high risk for cervical cancer.

## Introduction

The incidence of invasive uterine cervical adenocarcinoma relative to squamous cell carcinoma appears to be increasing (1–4). Screening has led to early detection and successful treatment of intraepithelial squamous lesions. In contrast, however, there has been an overall rising incidence of adenocarcinoma in situ (AIS) without a comparable decline in subsequent invasive disease (5,6).

A number of clinical challenges arise in association with AIS. Firstly, on screening cytology there is a lower likelihood of detecting glandular lesions compared to identifying pre-cancerous lesions of squamous origin (5,7). Particular attention is needed to obtain adequate endocervical samples, since glandular lesions are generally high in the cervix or deep within glands (1). For the same reason, it is harder to identify AIS compared to high-grade squamous intraepithelial lesions (HSIL) at colposcopy (8).

Liquid-based cytology (LBC) may help increase the accuracy of diagnosing glandular abnormalities, since a monolayer of cells is provided with reduction in interpretation errors (9). Nevertheless, cervical cytology has mainly been aimed at screening for squamous intraepithelial lesions and squamous cell carcinoma. Current approaches for handling glandular abnormalities, notably, ‘atypical glandular cells’ (AGC) found on cytology appear to be less than optimally effective in preventing cervical cancer (10,11).

Further along in the trajectory, namely once AIS has developed/been clinically detected, progression to invasive adenocarcinoma is reportedly more rapid compared to in situ squamous lesions. This may be due to the presence of multifocal disease with so-called ‘skip lesions’, i.e. non-contiguous foci of adenocarcinoma cells that increase the risk of residual/recurrent disease even when the excisional margins are clear (6).

For practically all aspects of cervical cancer development, high-risk human papilloma virus (HPV) is well established as the major contributor. Consequently, testing for HPV is essential for every facet of cervical cancer risk assessment. Included therein is HPV testing to estimate the chances of recurrent disease in patients who have been treated for intraepithelial cervical neoplasia (12–17). Assessment of HPV has been shown to be particularly valuable for patients with glandular abnormalities on cytology. A significantly higher percentage of HPV positivity was reported in 53 patients with AGC on LBC compared to 338 patients with cytology specimens negative for intraepithelial lesions or malignancy (NILM) (18). A more recent study which included Stockholm as well as other regions of Sweden, concordantly revealed that HPV triaging provided high predictive value and sensitivity for identifying patients with AGC who had cervical intra-epithelial neoplasia grade 2 (CIN2+) or worse (11).

With regard to risk of invasive cervical cancers, subtypes HPV16 and HPV18 have been most frequently identified (19). In our nine-year nested case-control follow-up study of patients with NILM at baseline, positive HPV16 and/or HPV18 were significantly associated with future risk of CIN2+. Among women younger than 30 at baseline, it was only the HPV16 or HPV18 subtypes that were linked to increased risk of CIN2+ (20). Both HPV16 and HPV18 have been the most frequently found subtypes in patients with AGC on cytology (21). These two subtypes were also significantly more prevalent among patients with AGC (20.8%) compared to 1.2% in controls with NILM (18).

As reviewed in Andersson et al (9), comparisons of the prevalence of the two subtypes, HPV16 and 18, indicate that HPV18 is less common than HPV16 among healthy women in their thirties, and also in patients with squamous carcinoma. On the other hand, HPV18 appears to be equally or even more prevalent than HPV16 in cervical adenocarcinoma. A local tropism for HPV18 in glandular cervical epithelium has been suggested as the mechanism for this finding. Of critical clinical importance, HPV18 is associated with increased risk of cervical adenocarcinoma, particularly with a more aggressive course (9,22). A recent population-based investigation from the U.S. (23) reveals that HPV16 was the most frequently detected subtype in AIS, as well as in CIN3, while HPV18 was the 2nd most often identified subtype in AIS, but was less frequently found in CIN3. Cleveland et al (23) underscore that AIS was more likely to be positive for the HPV subtypes targeted in vaccines. They note a significant decrease in AIS incidence among women aged 21–24, likely reflecting the effectiveness of the vaccines vis-à-vis these two HPV subtypes in younger women.

With a focus on management and surveillance of AIS, particularly when conservative treatment is preferred, as is very often the case for women during their reproductive years, Teoh et al (6) have recently described the specific clinical guidelines of the Society of Gynecologic Oncology, as reviewed and endorsed by the American Society of Colposcopy and Cervical Pathology. These authors emphasize that such guidelines have been heretofore lacking, and base their recommendations on the state-of-the-art knowledge, albeit often limited. Among their recommendations are rigorously regular cytologic and HPV co-testing, especially for HPV16 and HPV18, as well as endocervical sampling, as semi-annual follow-up for at least three years post-excision, insofar as the excisional margins were clear. Thereafter, if co-testing and endocervical sampling have been consistently normal, this interval can be lengthened to annually during the first five years post-excision, after which they consider that the surveillance interval can be safely extended to triennially. However, insofar the excisional margins were not clear, re-excision is recommended if feasible and safe. If the re-excision yields clear margins, then the above-described suggested protocol can be implemented.

The aim of the present study is to provide a detailed review of a cohort of patients first time treated by conization for histologically-confirmed AIS. Our primary goal is to assess the risk factors associated with recurrent/persistent AIS, as well as any progression to microinvasive or invasive cervical cancer. The present study is multi-faceted. It is informed by the above-described guidelines (6), together with our previous experience in evaluating the predictors of recurrent high-grade cervical dysplasia in a cohort of patients followed for up to six years post-conization, and who had primarily squamous pathology but also with some cases of AIS (24). Besides the current focus exclusively on patients treated for AIS, the present study provides a much longer follow-up period than the previous study (24), starting from the year 2001 up to the present. We aim to identify the predictors of recurrent AIS in this cohort of patients. We also critically evaluate the adequacy of follow-up in order to suggest practical improvements that could better protect this high-risk cohort.

## Materials and methods

### Population under study and location

Patients who had undergone first-time conization from 2001 until January 2017 at Stockholm Hospitals: Karolinska University, Danderyd or South General with histopathologically-confirmed AIS in the excised cone were eligible to be included in the present study. The patients to be included in the present study were identified using the Swedish National Cervical Screening Registry, which contains complete cervical screening data for the entire country since 1995 (10).

The study was approved by Regional Ethics Committee in Stockholm, based at the Karolinska Institute, which determined that participant informed consent was not required (Dnr:168/03, 2004-679/3, 2010/944-32, 2013/763-32, 2014/2255-31/5, 2017-2007/32). Nevertheless, the option of blocking access to medical records was provided, such that women who had chosen to block access to their medical records would be excluded from the study. For the present study, none of the women blocked their records. Thus, there were no patients excluded as a result of blocking access to medical records.

Twenty-seven of the patients included in the present study were also included in an earlier follow-up study. From October 2014 until January 2017, patients first-time treated by conization for histologi-cally-confirmed AIS, as well as patients treated for high-grade intra-epithelial squamous lesions at Stockholm Hospitals: Karolinska University, Danderyd or South General also participated in an intensive follow-up study, as described previously (24,25). That study was approved by the Karolinska Ethics Committee study protocol (2006/1273-31, 2014/2034-3).

### Review of medical records

The complete medical records for each patient were exhaustively reviewed through April 2022. The year of the primary conization and the patient's age at that time were recorded, as were the modality of conization, histopathology and grade of dysplasia in the excised cone, and margin status in the cone biopsy. Clear margins were defined as having no high-grade dysplasia in the surgical margins and endocervical curettage, as usually performed post-conization in Sweden (26).

When reported, the patient's smoking status was noted. The categories were: currently smoking, having previously smoked and quit, or never having smoked. All comorbid diagnoses documented in the medical records were cited. Conditions that may interact with HPV acquisition or CIN progression were specifically noted. These include autoimmune disorders, malignancy, infection with hepatitis or human immunodeficiency virus, diabetes mellitus, genetic disorders or organ transplantation (26,27).

All post-conization follow-up data were fully examined. The number of months that had elapsed until the first gynecologic follow-up was documented, as well as the total number of post-conization gynecologic follow-up visits. Follow-up cytology results were reviewed, and categorized as all normal, or at least one abnormal result. The latter were classified as only low-grade versus high-grade (AIS or HSIL). Abnormal results were also classified as only glandular, only squamous, both glandular and squamous or undefined atypical cells. All HPV results post-conization were documented and summarized as follows: all negative HPV results, at least one positive HPV result, at least one positive HPV16 or HPV18 result, and two or more positive HPV results. We noted the total number of years of recorded gynecologic follow-up. The number of years that had elapsed without recorded gynecologic follow-up was also documented.

All gynecologic-related outcomes were documented. It was noted whether any reconization procedure had been performed. All hysterectomies and the reason for these were recorded. The most severe biopsy finding post-conization was noted. Patients with histopathologically-confirmed AIS, HSIL, microinvasive carcinoma or invasive disease were categorized has having confirmed detected recurrence. Patients with high-grade findings on cytology only (AIS or HSIL) without histopathology were classified as having likely recurrence but without histopathologic confirmation.

### Follow-up guidelines and procedures during the study period

During the earlier period of the study, Swedish National Cervical Cancer Guidelines for follow-up of patients treated for high-grade CIN were based upon margin status. Patients with negative margins underwent cytology after 6, 12, and 24 months and thereafter biennially. They were to be referred to colposcopy if any grade of dysplasia was present. Women with unclear or uncertain margins were to be followed up with colposcopy-directed biopsy and cytology within 4–6 months, or referred for reconization (26). More recently, HPV testing was included in the National Cervical Cancer Guidelines. After a follow-up gynecologic visit with negative HPV and NILM on cytology, the patients returned to routine triennial screening, as per National Guidelines.

During the earlier segment of the study, Papanicolaou (Pap) smears were employed. However, since the year 2010, the liquid-based method (ThinPrep^®^, Hologic, Marlborough, MA, USA) has been in use for cytologic analysis in Sweden.

The HPV tests in use at Karolinska at the time of the study were Cobas 4800 (Roche Molecular Diagnostics), Hybrid Capture 2 HPV DNA Test (Qiagen), and Linear Array HPV Genotyping Test (Roche Molecular Systems). Results were considered positive if any high-risk HPV: [16, 18, 31, 33, 35, 39, 45, 51, 52, 56, 58, 59, 68] or potentially high-risk: [26, 53, 66, 68, 73, 82] types were identified.

The patients first-time treated by conization for histologi-cally-confirmed AIS from October 2014 until January 2017, as part of the study described previously (24,25) also had clinician-taken cervical samples as well as vaginal self-collected and urine samples analyzed by the RealTime High-Risk HPV polymerase chain reaction assay of Abbott at first gynecologic follow-up.

### Statistical analysis

Firstly, comprehensive univariate and bivariate analyses were carried out. The latter was performed using 2-sample ‘t’ tests, Mann-Whitney (MW) tests, Pearson *χ*^2^ or Fisher's exact test if any expected cell was less than five. Unless explicitly stated otherwise, all comparisons were two-sided. The result of the MW test was cited whenever the continuous or semi-continuous variable deviated from a normal distribution (skewness and/or kurtosis ≥1.5). For significant bivariate associations with the outcome being detected recurrence of histopathologically-confirmed high-grade CIN, sensitivity and specificity were computed with 95% confidence intervals (CI), as well as negative predictive values (NPV) and positive predictive values (PPV). To compute odds ratios (OR) and 95% CI, multiple logistic regression (MLR) was employed, with the outcome being detected recurrence of histopathologically-confirmed high-grade CIN. For this statistical analysis, the 14.0.0.15 2020 TIBCO version of the Statistica software was used.

## Results

Altogether 84 patients were identified who had undergone primary conization at one of the above-named hospitals in Stockholm and in whom AIS was histopathologically confirmed in the excised cone. This includes the twenty-seven patients who participated in the studies as described previously (24,25).

### Baseline univariate data

As seen in Table I, the vast majority of the patients were 40 years of age or younger at the time of primary conization. Most of the patients had undergone conization up to the year 2015, with laser conization being the most frequent surgical technique. More often than not, coexisting squamous pathology together with AIS was found in the histology of the excised cone, with the highest grade, CIN3 being most common. Precisely half of the patients had incomplete excision of the lesion; in slightly over 25% of cases both margins were unclear or uncertain.

Smoking status was indicated in the medical records of relatively few of the patients, (36.9%). Among those for whom this information was available, over 70% of the patients had never smoked.

Fifty-four patients (64.3%) had one or more comorbid diagnosis reported in their medical records. Overall, the most frequent were psychiatric disorders in 15 patients (17.9%), among whom 13 patients were noted to have clinical depression. Over 20% of the patients had two or more diagnosed comorbidities. Fourteen patients (16.7%) had a comorbid diagnosis assumed to interact with HPV acquisition or CIN progression, among these were autoimmune disorders in eight patients. The autoimmune conditions included inflammatory bowel disease, autoimmune thyroiditis and multiple sclerosis. Two patients had diabetes mellitus. Of the four patients with a diagnosed malignancy, two patients had tonsillar cancer, one patient had breast cancer and one patient had lung cancer. Ten patients had other gynecologic diagnoses; these included endometriosis, ectopic pregnancy, ovarian cyst and infertility. Three patients had undergone endometrial biopsy.

### Follow-up univariate data

The follow-up data, as displayed in Table II, indicate that well over half of the patients had a first follow-up within six months of the primary conization and had three or more follow-up examinations. Nearly 60% of the patients had normal cytology on all the examinations subsequent to the primary conization. High-grade cytology (AIS and/or HSIL) was found in five patients. A total of five patients had no cytology results post-conization.

In sharp contrast, over 38% of the patients had no HPV results whatsoever during follow-up, while nearly 30% had only one reported HPV analysis post-conization. Altogether just over 20% of the patients had one or more positive HPV result, while positivity for the high risk subtypes 16 or 18 was reported in a total of ten patients. There were also ten patients in whom two or more HPV results were positive post-conization.

The mean number of years of follow-up was 4.6±3.6, with a maximum of 14 years. On the other hand, the mean number of years without recorded gynecologic follow-up was 5.3±3.9.

### Univariate analysis vis-à-vis outcomes

A total of 28, i.e. one-third of the patients, underwent another operation subsequent to primary conization (Table III). Reconization was performed in nearly 20% of the patients, while just over 20% underwent hysterectomy. An equal percent, nearly ten percent, of the patients underwent hysterectomy for unclear margins at initial conization and for residual or recurrent dysplasia. One patient was treated by hysterectomy for a positive HPV18 result recorded post-conization, although reconization biopsy revealed only adenomyosis with no evidence of recurrence.

Over 60% of the patients had no biopsy results post-conization. Among those with abnormal biopsy results, recurrent AIS was the most common histopathologic finding. In addition to the ten patients whose most severe reported biopsy findings were high-grade cervical intra-epithelial neoplasia, one patient was found to have microinvasive carcinoma together with AIS and another patient had invasive adenosquamous carcinoma. Altogether, twelve patients had recurrence of high-grade intra-epithelial neoplasia or worse at follow-up, confirmed by post-conization biopsy. In addition, there were three patients for whom residual/recurrent disease was likely, but without histopathologic confirmation. One of these patients had AIS/HSIL on cytology three years post-conization at which the margins were reportedly clear; she was treated shortly thereafter with hysterectomy. There was no reported histopathology from the hysterectomy, nor were there any other post-conization data on that patient. Another patient had AIS on cytology four months post-conization at which the margins were reportedly clear; she was treated with hysterectomy thereafter with no histopathologic findings reported. The only other post-conization data on that second patient were two normal vaginal cytology findings post-hysterectomy. The third patient also had AIS on cytology two months post-conization and immediately thereafter underwent hysterectomy with no histopathology reported. The endocervical margin was unclear at primary conization. Post-hysterectomy there were two normal vaginal cytology findings and two normal HPV findings in that third patient.

### Salient bivariate analysis

Age at conization showed no significant association with abnormal cytology at follow-up nor with margin status or overall comorbidity (MW test). However, patients with diagnosed comorbidity linked to HPV or CIN progression were significantly older (41.9±12.9) than the patients without these diagnosed comorbidities (33.6±6.9) (MW test z=2.6, P=0.01). Among the 52 patients with at least one HPV result, there were no significant differences in age for those with at least one positive result compared to those patients with negative HPV findings. Significantly more than the expected number of patients with one or both unclear or uncertain margins also had at least one abnormal cytology finding, (Pearson's χ^2^=9.5, P=0.002). Among the 51 patients with at least one HPV and cytology result, significantly more (eleven) than the expected number of patients (seven) had both a positive HPV finding and abnormal cytology (Pearson's χ^2^=5.8, P=0.016).

In all of the bivariate analyses vis-à-vis detected recurrence, we excluded the patients who had undergone hysterectomy for incomplete resection very soon after primary conization and those patients with likely recurrence/residual disease but for whom there were no confirmatory histopathologic findings. With those exclusions, there was no significant difference in age at conization for the patients with detected recurrence compared to those in whom recurrence had not been detected. There were no significant or near significant associations between any of the comorbidity variables and detected recurrence. Neither surgical method of conization nor the histology of the excised cone showed any relation with detected recurrence.

Detected recurrence was significantly more frequent among the patients with any unclear or uncertain margin (Pearson χ^2^=6.7, P=0.01). There were borderline significantly more than expected detected recurrences for unclear or uncertain endocervical margin (Fisher's exact test, one tailed P=0.05). However, neither ectocervical margin alone nor both margins being unclear or uncertain were significantly associated with detected recurrence.

As noted from Table I, the smoking status was unknown in the majority of the patients. Among the patients for whom this information was available from the medical records, significantly more than the statistically expected number of patients with detected recurrence were currently smoking or had previously smoked and quit. Namely, five of the patients with detected recurrence were either currently smoking or had previously smoked (1.7 was the expected number) while two of the patients with detected recurrence had never smoked (5.3 was the expected number), Fisher's exact test P=0.0022.

As could be anticipated, significantly more (eight) than the statistically expected number (3.7) of patients with detected recurrence had at least one abnormal post-conization cytology finding (patients with no cytology results were excluded from this analysis). The analysis yielded a Fisher's exact test P=0.004.

Among the patients for whom there was at least one HPV result, several significant findings were noted vis-à-vis detected recurrence. While 2.7 was the statistically expected number of detected recurrences with a positive HPV finding, seven of the patients with detected recurrence had at least one positive HPV result (Fisher's exact test P=0.001). Six of the patients with an HPV18 positive result were detected to have recurrence, whereas 1.2 was the statistically expected number (Fisher's exact test P=0.000).

There were 2.4 expected recurrences for patients with two or more positive HPV results. However, six patients with two or more positive HPV results had a detected recurrence (Fisher's exact test P=0.002) (In this analysis only patients with at least two HPV tests were included).

The mean number of post-conization HPV results was greater among the patients with detected recurrence (2.9±2.8) versus without detected recurrence (1.2±1.3) (t=3.2, P=0.002). The number of years of follow-up was also higher among the patients with detected recurrence, 6.8±3.6, compared to 4.1±3.4 among the patients without detected recurrence (t=2.5, P=0.02).

### Assessments of sensitivity, specificity, negative and positive predictive value for selected significant factors in bivariate analysis with detected recurrence as the outcome

Table IV displays the results of our computations of sensitivity, specificity, negative and positive predictive value, as well as overall accuracy for several of the independent variables that were significantly associated with detected recurrence, as presented in the subsection immediately above. The exclusions for each of those independent variables in Table IV were as follows: For abnormal cytology at follow up, patients were excluded who underwent hysterectomy shortly after primary conization for incomplete resection or had no histopathological confirmation of likely recurrent/residual disease and/or had no follow-up data for cytology (N=71). For having one or more HPV findings at follow-up, 47 patients were included. Patients were excluded who underwent hysterectomy shortly after primary conization for incomplete resection, or had no histopathological confirmation of likely recurrent/residual disease or had no follow-up data for HPV. Twenty-six patients were included for persistent HPV-positive at follow-up, defined as two or more HPV positive results post-conization. Patients were excluded who underwent hysterectomy shortly after primary conization for incomplete resection or had no histopathological confirmation of likely recurrent/residual disease or had no more than 1 HPV follow-up finding. Altogether 74 patients were included for any unclear or uncertain margin; patients were excluded who underwent hysterectomy shortly after primary conization for incomplete resection or had no histopathologic confirmation of likely recurrent/residual disease. For currently or previously having smoked, 25 patients were included. Patients were excluded who underwent hysterectomy shortly after primary conization for incomplete resection or had no histopathologic confirmation of likely recurrent/residual disease or for whom there were no data in their medical records regarding smoking.

Albeit with a markedly small number of included patients due to lack of HPV data, it is seen that having at least one positive HPV finding at follow-up showed the highest sensitivity for detected recurrence. Currently smoking or having previously smoked provided the highest specificity for detected recurrence. However, the sensitivity was the lowest with the widest confidence intervals for that variable. As a reflection of the larger number of patients included, the CI's were the narrowest for any unclear or uncertain margin. Most notable was the low specificity of unclear or uncertain margins vis-à-vis detected recurrence.

### Multiple logistic regression findings with detected recurrence as the outcome

In Table V four significant multiple logistic regression, MLR, models are presented. For each of the models, detected recurrence of high-grade CIN or worse is the outcome. Patients who underwent hysterectomy shortly after primary conization for incomplete resection or who had no histopathologic confirmation of likely recurrent disease were excluded from all four models. Forty-seven patients were included in the top two MLR models since patients without any cytology and/or HPV findings were also excluded. In the third model, patients were excluded who had no follow-up data for cytology and/or had no more than 1 HPV follow-up finding.

The most powerful model (topmost) shows that adjusting for age at conization and abnormal cytology at follow-up, a positive HPV18 finding was the strongest and most significant predictor of detected recurrence. Albeit somewhat less powerful and also with a very wide 95% confidence interval, one or more positive HPV findings was also a significant predictor of detected recurrence, adjusting for age and abnormal cytology at follow-up (2nd model from the top).

The third MLR model required a more stringent exclusion vis-à-vis the HPV results, such that only patients with two or more HPV findings were included. Among those 26 patients, persistent HPV positive findings, i.e. ≥2 positive HPV results, were significantly associated with detected recurrence, adjusting for abnormal cytology at follow-up and age at conization.

Finally, on the bottom panel of Table V is an MLR model with the only exclusions being patients who underwent hysterectomy soon after primary conization or who had no histopathologic confirmation of likely recurrent/residual disease. Adjusting for age at conization, patients with any unclear or uncertain margin were over seven times more likely to have detected recurrence compared to patients with clear margins. This finding, however, became statistically non-significant when abnormal cytology at follow-up was included in the MLR model.

## Discussion

With up to fourteen years of recorded follow-up, the most powerful findings of the present study are the value of high-risk human papillomavirus, HPV, in predicting detected recurrence of high-grade intra-epithelial neoplasia or worse, among patients with glandular pathology at primary conization. This is particularly evident with regard to HPV18. Persistent HPV positivity at follow-up is also a powerful multivariate predictor of detected recurrence. It is therefore of concern that nearly forty percent of these patients had no HPV results whatsoever during follow-up, and another nearly thirty percent of patients had only a single HPV result post-conization. In sharp contrast, nearly eighty percent of the patients had two or more follow-up examinations and altogether a small percent, five patients in total, had no cytology results post-conization. In other words, most of the patients were not adequately followed by HPV testing post-conization. Instead, most of the patients were followed primarily by cytologic examination, which according to the present findings, did not independently predict the cases of biopsy confirmed, detected recurrent disease.

Several studies examining the outcome of patients treated conservatively for cervical AIS have focused upon margin status (5,28–30). The question raised was whether local excision is sufficient to protect these patients in the long-term. As was found in our study, the risk of recurrence was consistently reported to be higher with unclear margins. However, recurrences have also been reported among patients treated for cervical AIS who had clear excisional margins. As concluded in the paper by Young and colleagues: ‘Even with negative conization margins, women [treated conservatively for AIS] still face a risk of residual, recurrent, or invasive disease’ p. 195.e1. In our study, two of the twelve patients with detected biopsy-confirmed recurrence had clear endocervical and ectocervical margins. For only one of those patients were HPV results available. In that patient, HPV18 positivity as well as persistent HPV were found.

Investigations of patients treated by conization for AIS from four Italian centers included consideration of baseline HPV at conization and at follow-up, as well as margin status (31,32). Similarly to our study, approximately half of the patients had all clear margins on primary conization. Unclear margin(s) (31,32) showed a significant univariate association with persistent or recurrent disease. Also similarly to our study, positive HPV was reported to significantly predict persistence/recurrence in these patients. In the earlier of the two studies (31) which included 42 patients at baseline, it was only at first 6-month follow-up that HPV findings were significantly associated with detected persistent/recurrent disease, also with a wide confidence interval similarly our findings. In the study by Costa et al (31) HPV18 was reportedly positive in eight of 19 tested patients, but it was not specified whether that result was from baseline or at follow-up. Overall, very sparse univariate data were presented vis-à-vis HPV (31,32). Further information, i.e. the number of post-conization HPV positive results, persistent HPV positivity *inter alia* would be of interest.

Margin status as well as post-conization HPV have also been examined with regard to disease recurrence in a study of 701 patients with high-grade CIN, AIS or microinvasive cervical disease (33). Ten percent of the available HPV results were positive and showed a significant, multivariate association with recurrence with a wide confidence interval, as in our study. Unfortunately, no stratified analysis for the patients with glandular disease was reported regarding the predictive value of post-conization HPV. A more recent publication (34) provides a systematic review and meta-analysis of studies examining incomplete excision as well as HPV as predictors of treatment failure for cervical precancer. Although the relatively greater danger associated with glandular pathology was noted (34), no stratified meta-analysis of the data for patients with AIS was given.

Another possible risk factor with regard to recurrence was assessed in 71 patients treated by conization for in situ glandular pathology (35). Namely, does the risk of recurrence differ for AIS alone (forty-one patients) versus AIS with coexisting squamous pathology (thirty patients)? With a median follow-up of nearly five years, recurrence was found to be significantly higher among the patients with AIS alone. The results from Song et al (35) differ from our study, which, as noted, showed no significant relation between cone histology and detected histopathologically-confirmed recurrence.

Albeit without sufficient power for multivariate analysis due to the preponderance of missing data, our statistically significant bivariate finding was that patients who were currently smoking or had previously smoked were more likely to have a detected recurrence compared to non-smokers. In the meta-analysis of 1,278 patients treated by conization for AIS, a mean of 35% with a range of 20 to 57% of the patients were reported to be smokers (5). In a more recent study (28) these data were reported for over 80% of the patients treated for AIS, 31% of whom were currently smoking or had previously smoked. However, no analysis was provided as to whether or not smoking was associated with risk of post-conization recurrence for patients with *in situ* glandular pathology. On the other hand, a case-control study (36) of treatment failure among women treated for CIN indicated a three-fold higher risk of treatment failure for those who were currently smoking compared to patients treated for CIN who had never smoked (univariate analysis). This finding remained significant when adjusting for post-treatment HPV infection. Moreover, a dose-response effect was observed, with a concomitant rise in univariate and adjusted risk of treatment failure. The patients who currently smoked 30 cigarettes per day were at nearly eighteen-fold higher risk of treatment failure compared to patients who had never-smoked. The cervical pathology of the patients included in the investigation of Acladious et al (36) was described as CIN, without specification as to whether this was squamous or glandular. The concluding statement in Acladious et al (36) p. 438 was: ‘Women should be more aware of the hazards smoking presents in relation to cervical cancer. Smokers should be encouraged to stop smoking after treatment for CIN’. The results of our study fully cohere with that statement, and indicate that attention to smoking should be a routine component of risk assessment and treatment for patients with adenocarcinoma-*in-situ*.

Besides smoking and margin status at conization, post-conization HPV is unequivocally shown in the present study to be a significant, independent predictor of detected recurrent/residual disease in patients treated for AIS, irrespective of coexistent squamous pathology. Unfortunately, as stated, the majority of these patients have not been sufficiently followed post-conization with regard to HPV.

Of particular note is that a relatively small percentage of patients in the present cohort appear to have had gynecologic follow-up within the most recent period. This finding temporally coincides with the global COVID-19 pandemic, during which there has been a worldwide drop in cervical screening (37–41) with major deleterious consequences vis-à-vis cervical cancer incidence, treatment delay and mortality (39,42,43). During the 1st wave of the COVID pandemic, nearly 200 000 cervical screening appointments were cancelled in Stockholm (44). Numerous authors have suggested self-sampling for HPV as a viable cervical screening option, especially in face of the pandemic (37,41,44–48). Overall, self-sampling for HPV has been shown to be reliable (25,49–52), cost-effective (53) and acceptable among diverse populations, including women who are under-screened (54–59).

Our post-conization follow-up investigation of 479 women treated for high-grade CIN (60) indicates a high level of acceptability for HPV self-sampling. Notably, confidence in its reliability was a significant, independent predictor of willingness to perform HPV self-sampling (60). This concern is particularly germane for the present cohort. Namely, in our recent most studies (24,25) of patients treated by conization for high-grade CIN, vaginal self-sampling (VSS) for HPV showed overall high concordance with clinician sampling and high sensitivity for predicting recurrence among the patients with squamous pathology. However, VSS was found to be negative in all four of the patients with detected recurrent glandular pathology, whereas HPV was found to be positive in two of the four patients with standard Cobas clinician sampling and in three of the four patients with the Abbott clinician sampling. Further analysis in Andersson et al (24) of the 27 patients without detected recurrence in whom there was glandular histology in the excised cone and/or AGC on cytology at follow-up revealed that three patients showed HPV positivity on Abbott clinician-taken samples as well as VSS, in 22 patients both were HPV negative, one patient showed HPV positivity only on VSS and in one patient only the clinician sample was positive. Comparing VSS and Cobas clinician-taken samples showed similar findings, except that there were 23 patients with HPV negative findings from both methods, and in no case was Cobas positive when VSS was negative. On the basis of these results from Andersson et al (24), we suggested that VSS may not be inferior to clinician-sampling for follow-up of patients with glandular pathology. We underscored the need for further examination of this issue. Such investigation becomes that much more vital in light of the results of the present study, showing that the vast majority of the patients treated for AIS have been under-screened post-conization for HPV, which is an essential indicator of disease recurrence risk.

In the present cohort of patients treated for AIS, well over the majority had at least one diagnosed comorbidity according to their medical records. Fourteen of the 84 patients had a diagnosed comorbidity linked to HPV or CIN progression. Percentually, these figures are higher compared to our previous studies (24,26) of patients first-time treated for high-grade CIN, most of whom had squamous pathology. However, we found no relation whatsoever between comorbidity and detected recurrent disease in this study. Altogether two of the patients with histologically-confirmed recurrence had a diagnosed comorbidity linked to HPV or CIN progression, in one case an autoimmune disorder and in the other case diabetes mellitus. We thus concur with the conclusions from Andersson et al (24), that in individual cases these disorders may have been contributory and therefore relevant comorbidity warrants attention in clinical decision-making. That nearly 20% of the patients had a diagnosed psychiatric disorder, most frequently depression, also needs to be considered.

The vast majority of the present cohort was still within the potentially reproductive years at the time of conization. Altogether just over 20% of patients had undergone hysterectomy; those patients were significantly older at the time of conization (42.2±7.9) compared to those who had not undergone hysterectomy (33.0±7.8) (t=4.4, P=0.000). Among the 18 patients who underwent hysterectomy, one patient in her early fifties was operated for incomplete excision shortly after conization. Microinvasive disease and AIS were found on histology; no HPV results were noted. Another patient in her early fifties at the time of conization, with a positive past smoking history, was found at hysterectomy six years post-conization to have invasive adenosquamous carcinoma. There were no post-conization HPV results until after the hysterectomy, when vaginal HPV was found to be negative. A third patient in her early fifties, who also had previously smoked, was operated for incomplete excision shortly after conization. She was subsequently diagnosed with tonsillar cancer. Nine years post-hysterectomy, positive HPV16 was found on the vaginal sample. The risk of progression to microinvasive or invasive cervical or extra-cervical cancer among this cohort is poignantly illustrated by this brief review of the clinical course of these three patients. The need for vigilant HPV surveillance, including vaginal sampling post–hysterectomy is underscored.

The present cohort is relatively small. Power limitations must be therefore taken into consideration. As noted, various methods for HPV analysis were in use during the time when these patients were followed. It would be assumed that all positive HPV16 or HPV18 findings would have been explicitly recorded in the patient's records. However, since the HPV results were considered positive if any potentially or actual high-risk HPV type was identified, the subtype may not have been consistently noted. By far greater is the limitation due to no or insufficient follow-up with any recorded HPV result whatsoever in a large percentage of these patients. For 34 of the 66 patients who had not undergone hysterectomy, five or more years had elapsed without any follow-up noted in their clinical records. This lack of follow-up is sharply discrepant to the guidelines of the Society of Gynecology Oncology. Namely, indefinite surveillance is recommended even for patients treated for AIS with clear excisional margins and consistently negative HPV testing who do not undergo hysterectomy after completion of childbearing (6).

Overall, it can be concluded that this cohort of patients has been under-screened, particularly in the most recent period. As per the recently developed guidelines of the Society of Gynecology Oncology (6), patients treated for AIS require rigorous, regular follow-up, bearing in mind that ‘AIS [is] a unique diagnosis whose management needs to be differentiated from the management of the more prevalent squamous cell dysplasia’ p. 869. The strong predictive value of HPV, particularly HPV18 and persistent HPV positive results vis-à-vis detected recurrent cases in the present study indicates that regular HPV testing for patients treated for AIS is imperative. Investigations are urgently needed to determine whether HPV self-sampling could be a reliable option for patients with glandular cervical pathology. Furthering a participatory approach, including attention to smoking as a modifiable risk factor, together with strong encouragement to attend the needed long-term follow-up, offers hope to better protect this cohort of patients at high risk for cervical cancer.

## Figures and Tables

**Table I.** Baseline data for patients treated by conization for high-grade AIS.

Variable	No. patients	Percentage (%)
Age at time of conization		
21-30	28	33.3
31-40	39	46.4
41-50	10	11.9
51 or above	7	8.3
Year of conization		
2001-2005	5	6.0
2006-2010	36	42.9
2011-2015	30	35.7
2016	13	15.5
Surgical method		
C-LETZ	32	38.1
Laser	52	61.9
Histology of the excised cone		
AIS alone	34	40.5
AIS and coexisting squamous pathology	50	59.5
CIN1	4	4.8
CIN2	8	9.5
CIN3	38	45.2
Margin excision status		
Both margins clear	42	50.0
Only ectocervical margin unclear/uncertain	8	9.5
Only endocervical margin unclear/uncertain	12	14.3
Both margins unclear/uncertain	22	26.2
Smoking status		
Current smoker	7	8.3
Ex-smoker	2	2.4
Never smoker	22	26.2
Unknown smoking status	53	63.1
Any diagnosed comorbidity		
No	30	35.7
Yes	54	64.3
Two or more diagnosed comorbidities		
No	66	78.6
Yes	18	21.4
Diagnosed comorbidity linked to HPV or CIN progression		
No	70	83.3
Yes	14	16.7
Diagnosed comorbid malignancy		
No	80	95.2
Yes	4	4.8

CIN, cervical intraepithelial neoplasia; C-LETZ, contoured-loop excision of the transformation zone; HPV, high-risk human papillomavirus; AIS, adenocarcinoma-*in-situ*.

**Table II.** Follow-up of patients treated by conization for high-grade AIS.

Variable	No. patients	Percentage (%)
Time to first gynecological follow-up^a^		
Up to 6 months	45	54.9
7-11 months	25	30.5
1-2 years	4	4.9
Over two years	8	9.8
Number of follow-up examinations post-conization		
None	2	2.4
One	16	19.1
Two	18	21.4
Three or more	48	57.1
Cytology		
All normal cytology	49	58.3
At least one abnormal cytology	27	32.1
High-grade (HSIL, AIS)	5	6.0
Low-grade only	22	26.2
Glandular only	11	13.1
Squamous only	12	14.3
Glandular and squamous	2	2.4
Undefined atypical cells	2	2.4
No cytology results post-conization	5	6.0
Insufficient sample-no glandular epithelium	3	3.6
HPV		
Only negative HPV result(s)	34	40.5
At least one positive HPV result	18	21.4
HPV 16-positive	3	3.6
HPV 18-positive	7	8.3
Two or more positive HPV results	10	11.9
No HPV results post-conization	32	38.1
Only one HPV result post-conization	24	28.6
Number of years of recorded gynecologic follow-up		
≤2 years	30	35.7
2.1-6 years	30	35.7
6.1-10 years	18	21.4
>10 years	6	7.1
Number of years without recorded gynecologic follow-up		
≤2 years	25	29.8
2.1-6 years	27	32.1
6.1-10 years	23	27.4
>10 years	9	10.7

a The two patients without post-conization follow-up are excluded. AIS, adenocarcinoma *in-situ*; HPV, high-risk human papillomavirus; HSIL, high-grade squamous intraepithelial lesions.

**Table III.** Outcomes of patients treated by conization for high-grade AIS.

Variable	No. patients	Percentage (%)
Any reoperation		
No	56	66.7
Yes	28	33.3
Reconization		
No	68	80.9
Yes	16	19.1
Hysterectomy		
No	66	78.6
Yes	18	21.4
Reason for hysterectomy		
Unclear margin(s) in initial conization	8	9.5
Residual/recurrent dysplasia histopathologically confirmed	6	7.1
Likely residual/recurrent dysplasia but no histopathological confirmation	2	2.4
Positive HPV18 without evidence of recurrence	1	1.2
Other reason without evidence of recurrence	1	1.2
Most severe reported biopsy finding after 1st conization		
All normal findings	14	16.7
CIN1	2	2.4
CIN3	1	1.2
AIS	7	8.3
AIS and CIN	2	2.4
AIS and microinvasive carcinoma	1	1.2
Invasive adenosquamous carcinoma	1	1.2
Other (inflammation, adenomyosis, reactive changes)	5	6.0
Biopsy not done or results not reported	51	60.7
Detected recurrence		
No	69	82.1
Yes, histopathologically confirmed	12	14.3
Likely, but without histopathologic confirmation^a^	3	3.6

a See the main text for further details about these three patients. AIS, adenocarcinoma *in-situ*; CIN, cervical intraepithelial neoplasia; HPV, high-risk human papillomavirus.

**Table IV.** Sensitivity, specificity, negative and positive prediction of significant factors in bivariate analysis vis-à-vis outcome: Histopathologically confirmed recurrent/residual high-grade cervical intraepithelial neoplasia or worse in patients treated by conization for high-grade AIS.

Variable	NPV	PPV	Sensitivity (95% CI)	Specificity (95% CI)	Accuracy (95% CI)
Abnormal cytology at follow-up^a^	95.6	30.8	80.0 (44.4–97.5)	70.5 (57.4–81.5)	71.8 (59.9–81.9)
≥1 HPV-positive finding at follow-up^b^	96.8	43.8	87.5 (47.4–99.7)	76.9 (60.7–88.9)	78.7 (64.3–89.3)
≥2-HPV positive findings at follow-up^c^	94.1	66.7	85.7 (42.1–99.6)	84.2 (60.4–96.6)	84.6 (65.1–95.6)
Any margin unclear or uncertain^d^	95.0	26.5	81.8 (48.2–97.7)	60.3 (47.2–72.4)	63.5 (51.5–74.4)
Current or former smoker^e^	89.5	83.3	71.4 (29.0–96.3)	94.4 (72.7–99.9)	88.0 (68.8–97.5)

a N=71;

b N=47;

c N=26;

d N=74;

e N=25. See the main text for further details. CI, confidence intervals; HPV, high-risk human papillomavirus; NPV, negative predictive value; PPV, positive predictive value.

**Table V.** Multiple logistic regression for the outcome: Histopathologically confirmed recurrent/residual high-grade cervical intraepithelial neoplasia or worse in patients treated by conization for high-grade AIS.

A, Model χ^2^ =24.0 (P<0.001; N=47).				

Variable	OR	−95% CI	+95% CI	P-value
Age at conization	1.15	0.97	1.37	NS
Abnormal cytology at follow-up	1.36	0.07	24.9	NS
HPV18-positive finding at follow-up	141	5.2	3,803	<0.005

**B, Model χ^2^ =18.3 (P<0.001; N=47).**				

**Variable**	**OR**	**−95% CI**	**+95% CI**	**P-value**

Age at conization	1.19	0.99	1.43	NS
Abnormal cytology at follow-up	4.40	0.47	41.4	NS
≥1 HPV-positive finding at follow-up	47.6	1.77	1,283	<0.02

**C, Model χ^2^ =13.7 (P<0.01; N=26).**				

**Variable**	**OR**	**−95% CI**	**+95% CI**	**P-value**

Age at conization	1.21	0.90	1.63	NS
Abnormal cytology at follow-up	2.67	0.18	39.4	NS
≥2 HPV-positive findings at follow-up	89	1.91	4,141	<0.02

**D, Model χ^2^ =8.58 (P<0.02; N=74).**				

**Variable**	**OR**	**−95% CI**	**+95% CI**	**P-value**

Age at conization	1.05	0.98	1.13	NS
Any margin unclear or uncertain	7.21	1.34	38.7	<0.02

CI, confidence intervals; HPV, high-risk human papillomavirus; NS, statistically non-significant (P≥0.05); OR, odds ratio.

## Data Availability

The datasets used and/or analyzed during the current study are available from the corresponding author on reasonable request.

## Abbreviations

AGC: atypical glandular cells
AIS: adenocarcinoma-*in-situ*
CI: confidence intervals
CIN: cervical intraepithelial neoplasia
C-LETZ: contoured-loop excision of the transformation zone
HPV: high-risk human papillomavirus
HSIL: high-grade squamous intraepithelial lesions
LBC: liquid-based cytology
MLR: multiple logistic regression
MW: Mann-Whitney
NILM: negative for intraepithelial lesions or malignancy
NPV: negative predictive value
OR: odds ratio
NS: statistically non-significant
Pap: Papanicolaou
PPV: positive predictive value
VSS: vaginal self-sampling
